# A novel model of locking plate and locking spongious screw: a biomechanical in vitro comparison study with classical locking plate

**DOI:** 10.1186/s13018-024-04700-4

**Published:** 2024-04-12

**Authors:** Fatih Parmaksizoglu, Sinan Kilic, Onur Cetin

**Affiliations:** 1 Departmant of Orthopedics and Traumatology, Academic Hospital, Istanbul, Turkey; 2 Departmant of Orthopedics and Traumatology, NB Kadikoy Hospital, Istanbul, Turkey; 3 Departmant of Orthopedics and Traumatology, Istanbul Medipol University, Camlica Hospital, Istanbul, Turkey

**Keywords:** Locking plates, Screw failure, Plate fixation, Pull-out failure

## Abstract

**Background:**

Locking plates are commonly used for the fixation of comminuted, periprosthetic and osteoporotic bone fractures. These plates are secured to the bone with screws, creating a stable connection with fixed angle between the plate and the screws. In this biomechanical in vitro study, our aim is to evaluate and compare the novel locking plate-locking spongious screw model with FDA approved classical locking plate.

**Methods:**

Sawbone PCF-15 osteoporotic bone model was utilized to simulate osteoporotic bone conditions. Two screws were used to attach both the classical locking plate and the novel locking plate-locking spongious screw model to these bone models. The attachment strength of the screws to the bone blocks was measured by pull-out tests.

**Results:**

Novel locking plate-locking spongious screw model exhibited an 84.38% stronger attachment to the osteoporotic bone model compared to the current locking plate model.

**Conclusions:**

In conclusion, one of the important problems in the locking plates which is the high Pull-out risk of the locking spongious screws can been resolved with our proposed new model and has a chance of having a better purchase especially in osteoporotic bones.

## Background

After years of research, numerous aspects of bone fixation using the plate-screw system have become clear. To secure bone fragments effectively, axial, torsional, and bending forces must be counteracted [1].

Non-locking plates depends on friction forces between bone and plate to counteract shear forces. In contrast, locking plates convert shear stress to compressive stress at the screw-bone interface as a single beam construct. In addition, the sum of the holding strengths at all screw-bone interfaces equals the overall fixation strenght in locking plate construct, rather than depending on the frictional force generated by the screw compression as in non-locking plates [2, 3].

Especially in the last two decades, locking plates have gained increasing popularity and have a long learning curve and numerous failures since these constructs are relatively new implants [4]. The attachment of the plate to the bone rely on screws and various models have been developed to achieve fixed and rigid angle between plate-screw [5]. The locking plates act as an additional cortex and distribute loading forces at the fracture line equally to all screws at the same time [6].

In a sense, inspired by the external fixators used today, locking plates are described as internal-external fixators [7]. However, this plates provide better stability due to their proximity to the bone compared to the external fixator [6]. Locking plates offer a less rigid fixation system compared to non-locking plates, enabling stable motion that stimulates callus formation by secondary fracture healing among bone fragments [8, 9]. Especially fractures in osteoporotic bones, locking plates have better purchase and relatively lesser Pull-out risk than non-locking plates [4].

Rapidly growing industry gives orthopaedic surgeons various options in locking plates. Since the Pull-out problem -which is a major failure in osteoporotic bones- partially overcame with the invention of locking plates; however locking spongious screws are not an option in most of the FDA approved locking plates.

The hypothesis of our study is the usage of an technically proper locking spongious screws have better purchase and improves the Pull-out problem in osteoporotic bone models. As a result, we made a novel locking plate-locking spongious screw prototype.

The aim of this study is to evaluate and compare the pull-out strenght of our novel model of locking plate-locking spongious screw with an FDA approved classical locking plate in an osteoporotic bone model.

## Materials and methods

**Fig. 1:**
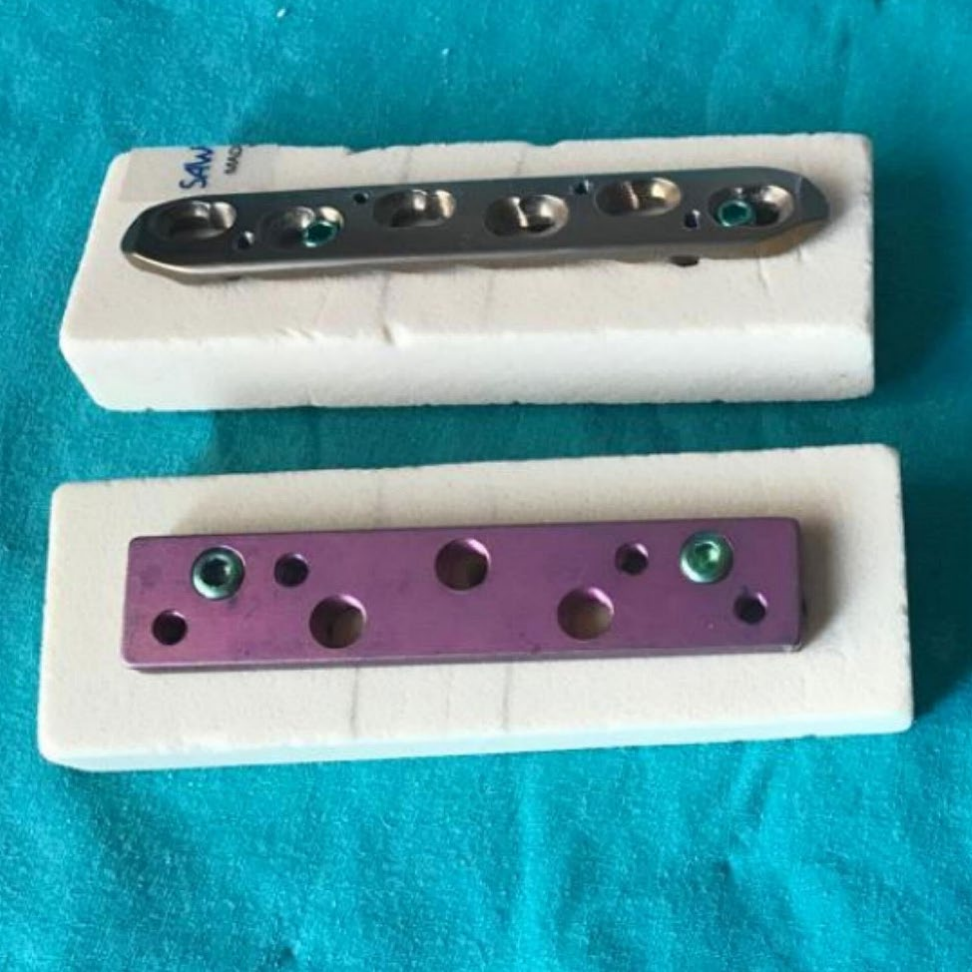
Osteoporotic bone model prepared with novel and classical locking plates fixed with 2 screws

**Fig. 2:**
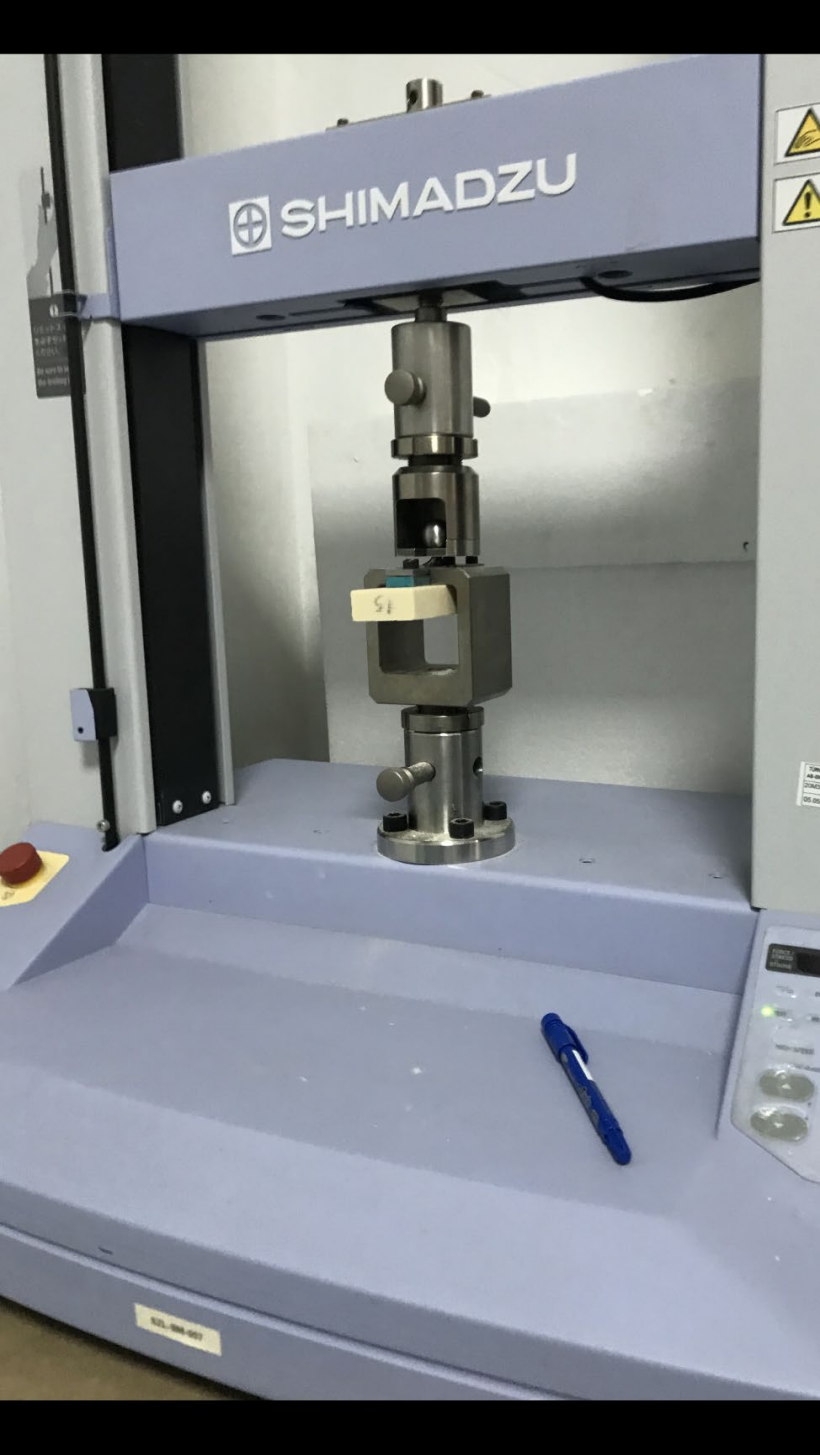
The Shimadzu AGS-X Pull-Out Device

This study compared the attachment strength to the osteoporotic bone of the classical locking plate-screw system (“LPS”, Merillife, Gujarat, India) and the novel locking plate-locking spongious screw. Seven pieces of the Sawbones PCF 15 artificial bone blocks (Pacific Research Laboratories, Vashon, Washington), each measuring 18 × 45 × 180 mm, were prepared for each group (Fig. 1). The Shimadzu AGS-X Pull-Out Device was used for the Pull-Out Tests (Fig. 2).

**Fig. 3:**
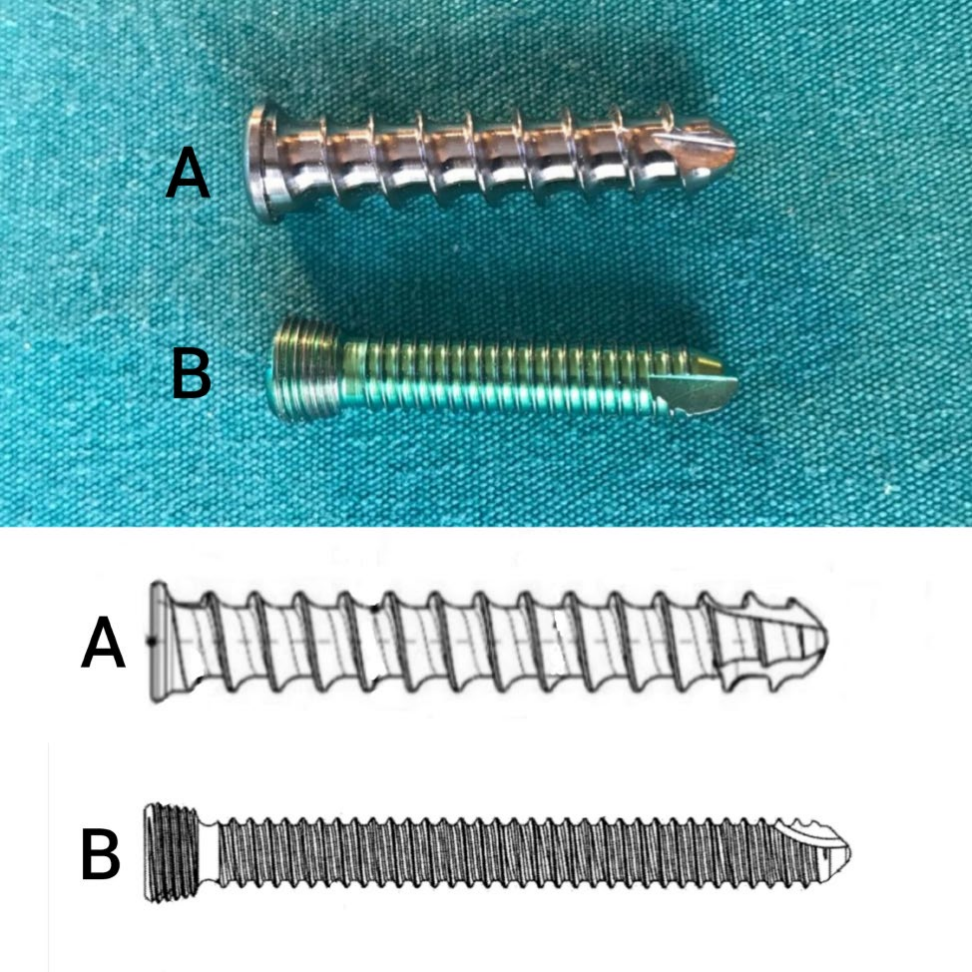
Novel Locking Spongious Screw Photography and Technical Drawing (**A**) and Classical Locking Screw Photography and Technical Drawing (**B**)

The novel plate features threads in the screw hole matching the threads of the new locking spongious screw model’s body, with consistent and same thread pitch distance throughout the screw’s length. There is no separate threads on the screw head, the screw is locked to the plate with the threads on its body from the beginning of the screwing (Fig. 3). The screw tip is designed for self-tapping and a hexagonal screwdriver slot is present on the screw head. The core diameter of the novel locking spongious screw is 4.6 mm, thread diameter is 6.6 mm thread pitch distance is 3 mm, thread depth is 1.0 mm. The novel plate and spongious screws material is made from titanium. The thickness of the novel plate is 5 mm and the width is 20 mm, and for the screw, drill bit diameter is 4.5 mm (Fig. 3).

For comparison, we aim to use a global firm’s FDA approved and widely used locking plate and screw (“LPS”, Merillife, Gujarat, India). LPS screw core diameter is 4.4 mm, thread diameter is 5.0 mm, thread pitch distance is 1 mm, thread depth is 0.3 mm. The thickness of LPS plate is 4 mm and the width is 17 mm, and for the screw, drill bit diameter is 4.3 mm (Fig. 3).

In both groups, the plates were centered on the Sawbones and secured with two locking screws at seven-centimeter intervals using a 3.0 N torque screwdriver (Fig. 1). Screw tips extended 5 mm from the other side, with the self-tapping ends of the screws protruding outside the Sawbone blocks.

A pulling apparatus which was a steel plate with an orbicular head was used in the test. Four new screw holes drilled specifically for this test on the classic locking plates for the pulling apparatus to attach on the locking plates. In consideration of potential plate breakage or damage to the original drill holes, smaller diameter drill holes were opened for the pulling apparatus on the original locking plate. In contrast, the novel plates were initially manufactured with larger drill holes.

**Fig. 4:**
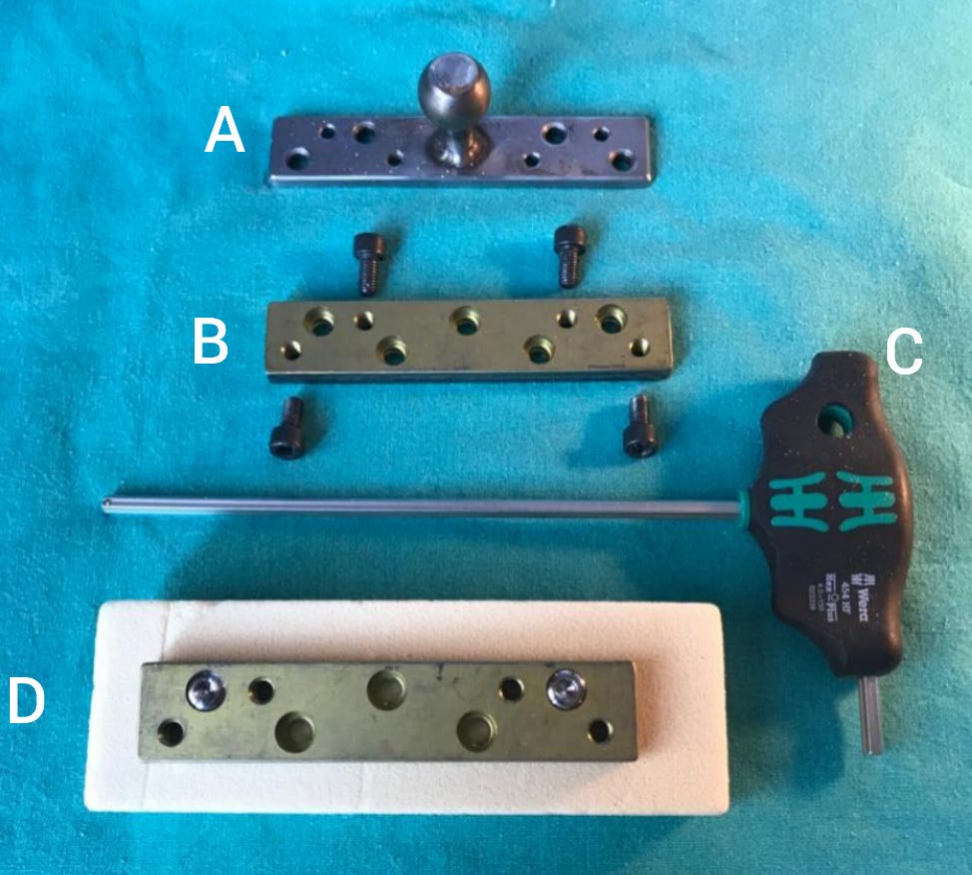
Pulling apparatus, a steel plate with orbicular head (**A**), novel plate and cylindirical socket screws prepared for pulling apparatus to fix on the plate (**B**), a hexagonal screwdriver (**C**), novel plate fixed with 2 spongious screw on the osteoporotic bone model (D)

For the test, pulling apparatus was placed on a fixated locking plate-sawbone system to assess the Pull-out force. Pulling apparatus fixed only to the locking plate with four steel screw which was small cylindrical head hexagon socket screws (Fig. 4). The purpose of these cylindrical head hexagon socket screw is to securely connect the pulling device to the plate. The different diameters of these cylindirical head hexagon socket screw had no effect on the result.

**Fig. 5:**
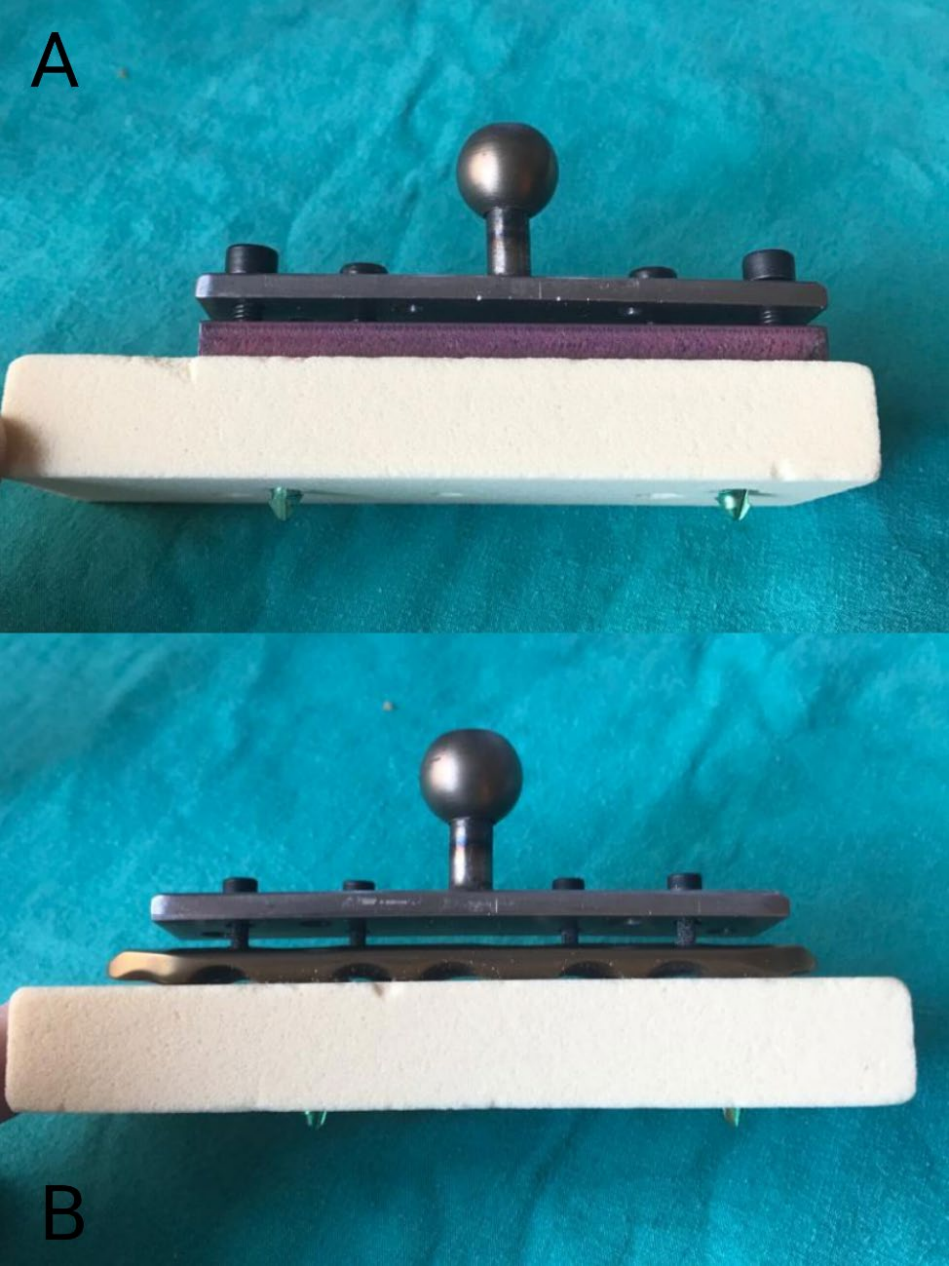
Pulling apparatus started screwing with 4 screws to the novel plate (**A**) and classical locking plate (**B**). Pulling apparatus will be fully seated on the plates after screwing completed

Consequently, the test materials took the form of a plate affixed to the Sawbone with two screws and a pulling apparatus fixed to the plate with four screws (Fig. 5). In the end, both plates and the pulling apparatus were prepared identically for the test.

**Fig. 6:**
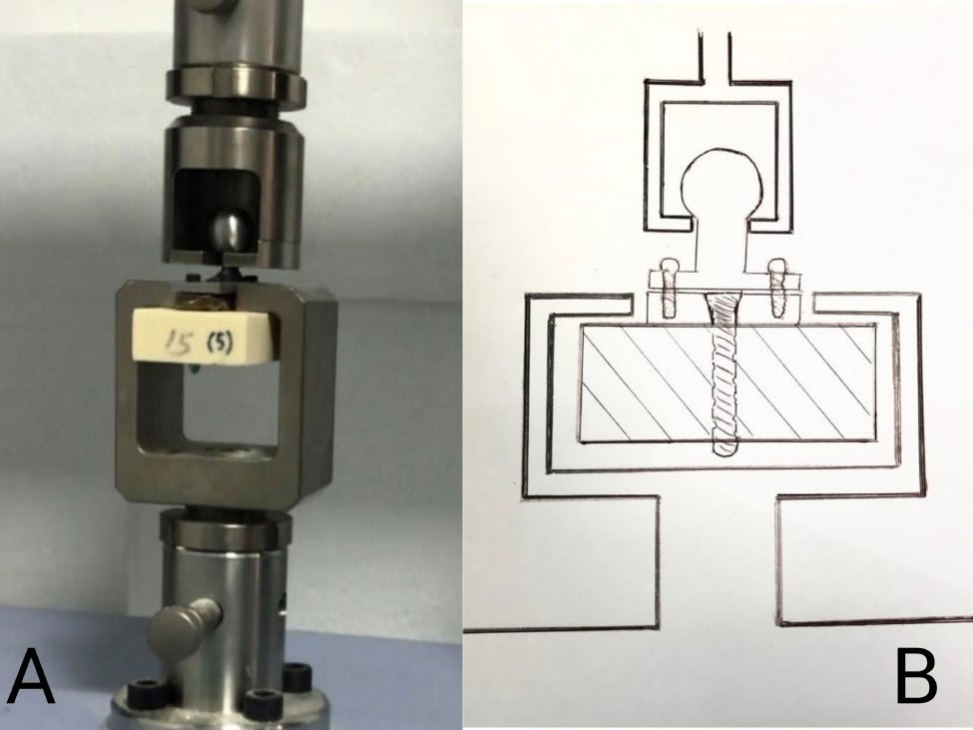
Plate and pulling apparatus in the Shimadzu Distraction Machine ready for test (**A**), and the illustration (**B**)

The prepared bone blocks with pulling apparatus were fixed to the holder in the lower part and the pulling apparatus was attached to the pulling part of the Shimadzu Distraction Machine and tests started with pulling force and force gradually increased (Fig. 6).

The force was incrementally increased until separation occurred. The pulling force was monitored graphically in Newtons on the monitor screen and the moment of separation of the screws were instantly detected. The test was repeated with seven blocks in each group that were prepared from each sample.

Descriptive statistics about the data in the study presented with mean and standard deviation values. With the use of Mann Whitney U test, the p value of < 0.05 was considered significant in the study.

## Results

**Fig. 7:**
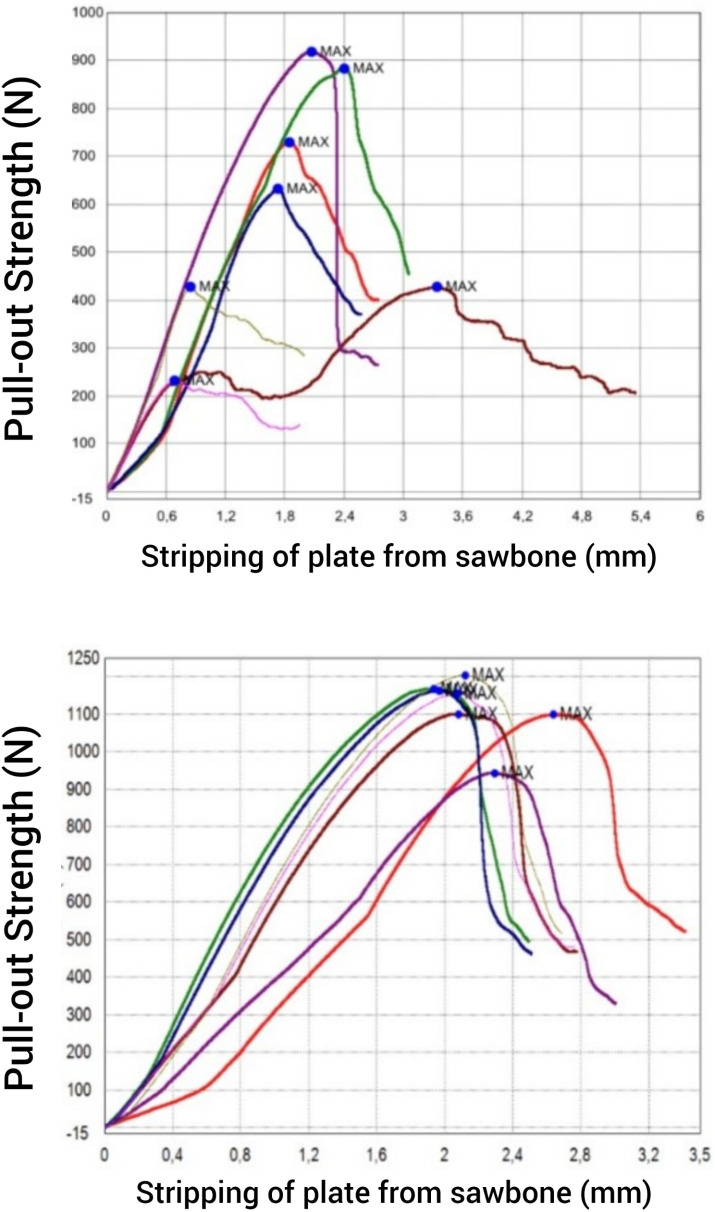
Maximum pull-out strength and stripping from sawbone of classical locking plates (Top) and novel locking plates (Bottom)

The stripping of the screw from the bone occurs with a minimum of 231.33 N and a maximum of 917.99 N in the classical locking plate model, with an average value of 606.82 N. In contrast, the stripping of the screw occurs with a minimum of 943.86 N and a maximum of 1203.83 N in the novel locking plate model, with an average value of 1118.91 N. According to this result, the novel locking plate-locking spongious screw model holds 84.38% stronger than the classical locking plate-screw system in the osteoporotic bone model. Statistical analysis using the Mann-Whitney U test further confirmed a statistically significant difference between the two groups (*P* = 0,009). Detailed results of both the classical and novel locking plates are presented in Fig. 7; Table 1.

**Table 1:** Test results of classical and novel locking plates

	Axial Pull-out Strength (N)	
Specimen	Classic Locking Plate	Novel Locking Plate
1	729,01	1098,54
2	426,79	1100,22
3	917,99	943,86
4	883,31	1167,94
5	632,40	1162,91
6	426,88	1203,83
7	231,33	1155,01
Average	606,82	1118,91
Standart deviation	256,46	85,88

## Discussion

Screw pull-out is a major cause of failure in osteoporotic bones and metaphyseal fractures, potentially leading to treatment failure and locking plates are developed to overcome this issue. However, spongious screws are typically not employed in a locking configuration when using locking plates [10]. Hypotetically, using locking spongious screws in osteoporotic bones may improve the fracture healing. In order to demonstrate the success of this combination, we aimed to develop a novel system that combines locking plates with locking spongious screw system and to compare it with an FDA-approved conventional locking plate-screw system in an osteoporotic bone model.

In non-locking plates, fixation accomplished with the friction force between the plate and the bone. As a result of rigid fixation, bone healing is achieved without callus formation (endosteal healing). In addition, excessive pressure of the plate on the bone may cause nutrition problems in the bone, which negatively affects healing and weakens resistance to infection [11].

In locking plates, shear forces converted into compressive forces at the screw-bone interface and the screws resist the deforming forces on the bone as a unified structure with forces distributed across all screws [6, 12].

Locking plates have minimal stretch which causes micro-movement at the fracture line. This micro-movement stimulates callus formation, resulting in secondary fracture healing (enchondral healing) [9, 13]. Micro-movement within locking plates should never occur at the screw-bone interface, as any micro-movement at this interface may exacerbate bone-screw connection problems leading to an unstable motion. In recent studies, it has been shown that a rigid fixation created by locking plates delays secondary fracture healing by creating callus [9, 12, 14]. This rate can reach 19% in periarticular fractures [15].

The key factor in the non-locking and locking plate-screw system is the attachment strength of the screws to the bone. If screws are firmly attached to the bone in both systems, the plate-screw fixation system can perform the expected function [10].

Failures in locking plates can be attributed to bone-related or implant-related causes [10]. Implant-related causes encompass plate bending, plate breakage, screw pull-out and screw head deficiencies [16]. Pull-out problem is a major cause of failure in locking plate applications [4, 10, 17]. New techniques in screw and locking plates can be promising in Pull-out problem such as hydroxyapatite-coated implants, polyaxial locking plates, far cortical locking, and improved screw design [18].

In classical locking plates, inadequate bonding to the porotic bone can easily occur, since the thread pitch distance and the depth of the threads are small. Studies conducted on treatments by using locking plates report a 20% suboptimal fracture healing [8]. When dealing with osteoporotic bone fixation, the use of locking spongious screws becoming more advantageous. Since the thread pitch distance and thread depth of the locking spongious screw are greater, it will attach to the porotic bone much better.

When locking commences promptly, the screw seamlessly advances within the bone, preserving the integrity of the attachment threads it establishes. In contrast, delayed locking results in potential damage to the attachment threads formed by the screw within the bone, leading to a weakened holding power in the bone-screw interface resulting an unstable micro-motion. This occurs as the screw undergoes rotation before successfully advancing into the bone. We can attribute the observed wide variation results in the holding power of screws within classical locking plates to this phenomenon.

Notably, the novel locking plates mitigate the possibility of such issues. Upon examining the holding forces of the novel locking screws, we observed close and stable values. This suggests a more reliable and consistent Pull-out performance compared to the potential challenges associated with the classical locking plates as a result of stable bone-screw interface and desired micro-motion.

In addition to these challenges, there are also minor issues with locking plates. Even if a guide hole is drilled using a sleeve in osteoporotic bone, the screw may shift to another angle during the screwing. If this axis slip angle is greater than 5^o^, the thread in the screw hole on the plate and the screw head will not align properly resulting in unsuccessful locking because of mismatch of the thread [16]. It becomes more intolerable in bigger diameters. It was demonstrated that 7.3 mm cannulated screws had early disengagement if the screw angulation is greater than 2^o^ [19].

In our novel locking plate system, a guide hole is created in the bone through the use of a sleeve and a drill bit suitable for the screw hole alignment on the plate. The screw’s threads are initially attached to the screw slot on the plate and then to the bone, preventing any deviation in the insertion angle. Screw threads pass through the slot on the plate and locking commences from the beginning of the screwing. This method ensures superior attachment to osteoporotic bone, even in metaphyseal regions where the cortex is thin.

As a result, the conducted in vitro Pull-out test results, the prototype we introduced have 84.38% better purchase and stable results in an osteoporotic bone model than conventional locking plate. We can attribute this result to the locking mechanisim of the spongious screws and initial locking with the first threads resulting a better thread in the bone with better purchase. Patients suffers osteoporosis with complicated fractures may have improved surgical results with higher union rates with this novel plate model as it may reduce one of the major locking plate failure reason of screw Pull-out and unstable micromovement.

Additionally, in this study, Pull-out tests were done with 2 screws in each plate-bone model. In case if all slots filled with screws, it becomes a single beam construct and affects the in vitro Pull-out results of screws. We tried to demonstrate the locking screws purchase and functioning fairly.

## Conclusion

In conclusion, one of the important problems in the locking plates which is the Pull-out risk of the locking spongious screws can be resolved with our proposed new model and has a chance of having a better purchase especially in osteoporotic bones.

The in vitro Pull-out tests demonstrated that the novel model exhibited significantly greater attachment strength than conventional locking plates. The study underscores the potential clinical significance of the novel fixation system in addressing challenges associated with screw Pull-out in osteoporotic bone scenarios, offering improved stability and strength.

Further studies should be conducted in vivo to demonstrate the clinical results with the hypotetically higher union rates as a result of stable micromotion in the bone-screw interface and potentional positive and negative results.

## Data Availability

The datasets used and/or analysed during the current study are available from the corresponding author on reasonable request (Dr Fatih Parmaksizoglu, drfatihpar@gmail.com).
